# Statistical Modelling of SPADs for Time-of-Flight LiDAR

**DOI:** 10.3390/s21134481

**Published:** 2021-06-30

**Authors:** Alfonso Incoronato, Mauro Locatelli, Franco Zappa

**Affiliations:** Dipartimento di Elettronica, Informazione e Bioingegneria, Politecnico di Milano, Piazza Leonardo da Vinci 32, I-20133 Milano, Italy; mauro2.locatelli@mail.polimi.it

**Keywords:** Single Photon Avalanche Diode (SPAD), Silicon Photo-Multipliers (SiPM), Light Detection and Ranging (Lidar), Time-of-Flight (TOF) measurements, Monte Carlo simulations

## Abstract

Time-of-Flight (TOF) based Light Detection and Ranging (LiDAR) is a widespread technique for distance measurements in both single-spot depth ranging and 3D mapping. Single Photon Avalanche Diode (SPAD) detectors provide single-photon sensitivity and allow in-pixel integration of a Time-to-Digital Converter (TDC) to measure the TOF of single-photons. From the repetitive acquisition of photons returning from multiple laser shots, it is possible to accumulate a TOF histogram, so as to identify the laser pulse return from unwelcome ambient light and compute the desired distance information. In order to properly predict the TOF histogram distribution and design each component of the LiDAR system, from SPAD to TDC and histogram processing, we present a detailed statistical modelling of the acquisition chain and we show the perfect matching with Monte Carlo simulations in very different operating conditions and very high background levels. We take into consideration SPAD non-idealities such as hold-off time, afterpulsing, and crosstalk, and we show the heavy pile-up distortion in case of high background. Moreover, we also model non-idealities of timing electronics chain, namely, TDC dead-time, limited number of storage cells for TOF data, and TDC sharing. Eventually, we show how the exploit the modelling to reversely extract the original LiDAR return signal from the distorted measured TOF data in different operating conditions.

## 1. Introduction

Light Detection and Ranging (LiDAR) is an optical technique widely used to measure the distance of a target and to acquire 3D depth-resolved maps of a scene, and is employed in several fields of science and everyday life, such as automotive applications [1], gesture recognition [2], 3D scanners for virtual prototyping [3], and security surveillance.

In Time-of-Flight (TOF) LIDAR, the target is illuminated with light and a camera collects backscattered photons to evaluate the distance of the target; depending on the light modulation, this can be performed in different ways [4,5]. The so-called ‘direct’ TOF, employs a pulsed laser and a time measurement circuit to directly measure laser pulse’s TOF and then the distance D from the object, according to D = ½∙TOF∙c, where c is the speed of light through the medium. In this approach, the TOF measurement jitter directly affects the distance precision: for 1 cm single-shot distance precision, the time measurement system must have uncertainty below 70 ps.

In many applications, eye-safety considerations place limits on the wavelength, optical power, and optical system (e.g., focal length, numerical aperture, etc.) used to illuminate the scene [6]; moreover, at long distances, light attenuation and beam divergence can reduce the light intensity that can be received by the TOF sensor. Therefore, Single Photon Avalanche Diode (SPAD) detectors and SPAD arrays can be employed as TOF imagers, thanks to their single-photon sensitivity and ability to integrate one Time-to-Digital Converter (TDC) in each pixel, allowing to reconstruct high frame rate 3D images [7]. Their gating capability can be exploited to image defined regions of interest in the distance plane, suppress direct leakage between illumination laser beam and sensor (e.g., when both are physically placed in the same enclosure), or even to suppress specific reflections from objects inside the scene, allowing for applications such as around-the-corner imaging [8], where the content of a scene hidden from direct line of sight can be reconstructed exploiting multiple reflections of a light beam. A drawback of using a SPAD in a LiDAR system is its extreme sensitivity, since it gets triggered by at least one photon, with no chance to signal how many concurrent photons composed the light pulse. This causes a strong susceptibility to background light (i.e., light naturally occurring in the environment, uncorrelated with the TOF illumination source), whose photons can saturate either the SPAD or the TDC conversions or even the electronics resources (e.g., TOF storage cells, digital readout speed, etc.), thus preventing the detection of just the desired signal.

A Silicon Photomultiplier (SiPM) is a solid-state single-photon detector which is constituted by many microcells all connected in parallel, for a single pixel detector. Each microcell consists in one SPAD and one passive quenching resistor, to provide a self-quenched analog current pulse when at least one photon hits the SPAD. Being the microcells all connected in parallel [9], the SiPM output consists of an analog current pulse, whose intensity is proportional to the number of concurrent photons hitting the SiPM (i.e., all microcells’ SPADs): for this reason, SiPM are photon-number resolved compared to single SPADs; hence, they allow to discriminate a bunch of signal photons (e.g., the laser pulse return) from few randomly arriving background ones [5]. Detailed electrical models of SPADs [10] and SiPMs [11] have already been reported for proper optimization of the front-end sensing electronics.

A single SPAD or a single SiPM can be used in single-spot LiDAR measurements, but also in 3D scene acquisitions by means of 2D scanning of the scene [5]. Instead, SPAD arrays and SiPM arrays (where each SiPM acts as an independent pixel) allow to acquire 3D scenes with no moving part, with a much shorter acquisition time. For example, Bronzi et al. [12] report a SPAD array where the independent SPAD pixels can be used in photon-counting for acquiring 3D scenes in automotive environment, but with no background rejection. In Ref. [13] an array of SPADs with multi-TDCs is proposed, to provide strong background rejection and localization of the laser spot across the array. Instead, in Ref. [14] Sony presented a complete LiDAR system based on a 189 × 600 pixels SPAD array, in a 3D-stacked technology, where each pixel is a microcell composed of 3 × 3 or 4 × 4 SPADs.

The proper design of a TOF LiDAR system, from the selection of the detector, to the array sizing, the design of electronics and optics, requires a deep understanding of the working operation and limitations of both the detector (being either a SPAD, a SiPM, or an array of them) and the TOF timing electronics, the TDC, the storage memory, and the post-processing. In order to provide a valuable tool for the sizing of these components and the simulation of the expected acquisition waveforms of signal and background photons, we have developed a detailed statistical modelling for SPAD-based LiDAR systems.

In this paper, we present the analytical statistical modelling and we compare the results with those of a Monte Carlo simulator in very different operating conditions with very high background levels. We show how the two approaches lead to perfectly matching results and can be interchangeably used to predict LiDAR systems performance and to select design choices for both the SPAD detectors and the TOF processing electronics. In particular, moving from Monte Carlo simulations to a compact and comprehensive statistical model drastically reduces the computational time and enables more and faster comparisons in different operating scenarios. In the statistical modelling of the SPAD detectors, we take into consideration non-idealities such as hold-off time, afterpulsing, and crosstalk, which cause a strong distortion of the measurements due to the pile-up of multiple photons. Moreover, we also take into consideration the non-idealities of the timing electronics chain, namely, the TDC dead-time, the limited number of storage cells for the TOF data, and the acquired TOF histogram, all of which cause the saturation of measurements when a strong ambient background overwhelms signal photons.

Beyond the statistical modelling of the SPAD, we also present how to model the acquisition electronics needed to suppress background, by discussing general-purpose methods such as coincident detection of multiple photons, time-gated detector operation, and multi-TDC architectures. Hence, we show how both modellings, starting from the actual LiDAR return waveform, can predict the final measured TOF histogram distribution in very different operating conditions. Eventually, we show how the reported modelling can be profitably exploited in an inverse way, starting from the acquired measurements, to reversely extract the original LiDAR return waveform, thus compensating for most distortions that could affect a real LiDAR system.

In Section 2 we present the statistical modelling of SPADs affected by all possible non-idealities, such as hold-off time, afterpulsing, and cross-talk. In Section 3 we model different acquisition techniques, such as for single- and multi-photon detections, coincident detections, time-gated approach and multi-TDCs architectures. Eventually, in Section 4 we correct the measured TOF data, by applying the modelling for both single-photon Time-Correlated Single Photon Counting setups and multi-photon acquisitions.

## 2. Statistical Modelling of the SPAD

At first, we start modelling SPAD arrays and digital SiPMs without taking into consideration the acquisition electronics and measurements technique. SPAD-based systems can experience saturations and distortions of measurements due to fact that they provide a digital pulse every time one photon (or more concurrent photons) hits the active area, triggers an avalanche current multiplication process [15], and gets sensed by a suitable quenching circuit, which provides a digital pulse [16]. Therefore, more photons hitting the SPAD at the same time provide just one pulse, such as if only one photon were detected. Moreover, after each triggering, the quenching circuit must quench the SPAD, wait a hold-off time, and then reset the SPAD back to operation (i.e., biased above its breakdown voltage); the overall minimum elapsed time between one triggering and the following potential one is called hold-off time or dead-time, during which the SPAD cannot detect other photons [17]. Both effects cause the so-called “pile-up” distortion in both photon-counting applications (i.e., when the actual number of incoming photons must be counted within a given frame time) and photon-timing acquisitions (i.e., when the actual waveform of an optical signal or multiple TOF measurements must be computed).

We start considering the case of non paralyzable dead-time, i.e., with a fixed duration from the first ignition, during which the SPAD is insensitive to photons: this is the way a classic active quenching circuit operates [16]. Then, we introduce other SPAD phenomena, such as afterpulsing (i.e., the probability to have a secondary avalanche ignition after a primary one, due to carriers trapping and subsequent release) and crosstalk (i.e., the probability to ignite a spurious avalanche due to the current flowing through another SPAD).

In order to compute the detection probability $P\left(DET_{xy}\left(t\right)\right)$ of each SPAD of the array in position x and y, we start from the incoming photon rate $\Phi_{\mathrm{in}}.$ The actual avalanche rate undergoes losses due to Photon Detection Probability (i.e., the product of quantum efficiency and avalanche triggering probability [15,17]) of SPADs, fill-factor (FF) and photon detection probability (PDP) of the imager: $\lambda_{\mathrm{detector}}\left(t\right)=\Phi_{\mathrm{in}}\left(t\right)\cdot \mathrm{FF}\cdot \mathrm{PDP}.$ Such a rate is usually composed by two contributions $\lambda_{\mathrm{detector}}\left(t\right)=\lambda_{\mathrm{signal}}\left(t\right)+\lambda_{\mathrm{noise}}\;\left(t\right)$: the former is given by the signal photon rate (usually concentrated in one or a few peaks), while the latter is given by background light and dark counts, which are assumed to be randomly scattered over time. Eventually, $\lambda_{\mathrm{detector}}$ is considered to be equally distributed among the SPADs of the array; hence, in each SPAD we have $\lambda_{\mathrm{spad}}\left(t\right)=\lambda_{\mathrm{detector}}\left(t\right)/N_{\mathrm{spad}}$. For the discrete-time approach, we employ a quantization time step $T_{\mathrm{step}}$ shorter than all transients involved in the measurements. Then, we normalize all time quantities with respect to such a step, e.g., $\Delta_{HO}=T_{HO}/T_{step}$).

In the following, each analytical estimation is cross-checked to MATLAB Monte Carlo simulations assumed as the golden reference when measurements are not available. Such simulations rely on the generation of random photon arrival times, according to Poisson statistics, and include all deterministic effects of SPAD detectors and related electronics. Finally, multiple repetitions of the laser shot and the frame acquisition are implemented, so as to improve the measurements Signal-to-Noise Ratio, at different levels of signal, background and dark counts, and to check the model in extremely different situations.

### 2.1. Hold-Off

Defects inside the active volume of SPADs can trap some charge during an avalanche current flow; the following carrier release can trigger a second avalanche injection, to give a spurious event (either count or TOF). To reduce such an afterpulsing effect, the SPAD has to be promptly quenched and kept below breakdown (so becoming blind) for a hold-off time, during which trapped charges get released without triggering any avalanche. The necessary hold-off time to almost empty traps depends on the fabrication technology and also on the quenching electronics. The SPAD dead-time is usually defined as the hold-off time plus the quenching and resetting time of the electronics. To distinguish the SPAD dead time from other dead-time sources (e.g., TDC), it will be called hold-off time (HO).

Now, let us consider the distortion caused by the hold-off time. The probability to have a detection event PH(k) at time index *k* = *t*/*T_step_* given an avalanche rate $\lambda_{spad}$ is:

$$P\left(PH\left(k\right)\right)=1-e^{-\lambda_{spad}\left(k\right)\cdot \;T_{step}}$$

This event corresponds to an actual detection DET(k) only if the SPAD is not in hold-off status ($\bar{HO}\left(k\right)$), without considering other avalanche triggering causes (e.g., afterpulsing and crosstalk). Thereby, detection probability is the intersection between the two independent events PH(k) and $\bar{HO}\left(k\right)$:

$$P(DET\left(\left(k\right)\right)=P\left(PH\left(k\right)\cap \;\bar{HO}\left(k\right)\right)=P\left(PH\left(k\right)\right)\cdot P\left(\;\bar{HO}\left(k\right)\right)$$

A SPAD is in hold-off condition at the time index k if in the previous $T_{HOLDOFF}$ time interval, at least another detection occurred, that is:

$$P\left(HO\left(k\right)\right)=P\left(\bar{\cap_{j=k-\Delta_{HO\;}}^{k-1}\bar{DET\left(k\right)}}\right)=P\left(\cup_{j=k-\Delta_{HO\;}}^{k-1}DET\left(j\right)\right)$$

Since all the j events are mutually exclusive in the interval $\left[\;k-\Delta_{HO\;},\;k-1\right]$ (there can be only one detection), the previous equation can be rewritten as:

$$P\left(\cup_{j=k-\Delta_{HO\;}}^{k-1}DET_{\;}\left(j\right)\right)=\sum_{j=k-\Delta_{HO\;}}^{k-1}P\left(DET\left(j\right)\right)$$

By imposing the initial conditions (e.g., by either turning on the SPAD at t = 0 or by assuming stationary conditions), the probability of having a detection is given by:

(1) $$P\left(DET\left(k\right)\right)=\left(1-e^{-\lambda_{spad}\left(k\right)\cdot \;T_{step}}\right)\cdot \left(1-\sum_{j=k-\Delta_{HO\;}}^{k-1}P\left(DET\left(j\right)\right)\right)$$

In stationary conditions, where the probability of having a detection is constant, this equation can be rewritten as *P*(*DET*(*k*)) = *P*(*DET*):

$$P\left(DET\right)=\frac{1-e^{-\lambda_{spad}\cdot \;T_{step}}}{1+\Delta_{HO\;}\cdot \left(1-e^{-\lambda_{spad}\cdot \;T_{step}}\right)}$$

The equivalent rate in the continuous-time domain is P(DET)/T_step_ for *T_step_* → 0, hence:

$$\lambda_{detection}=\underset{T_{step}\to 0}{\lim}\frac{1}{T_{step}}\cdot \frac{1-e^{-\lambda_{spad}\cdot \;T_{step}}}{1+\Delta_{HO\;}\cdot \left(1-e^{-\lambda_{spad}\cdot \;T_{step}}\right)}=\frac{\lambda_{spad}}{1+\lambda_{spad}\cdot T_{HOLDOFF}}$$

**Figure 1:**
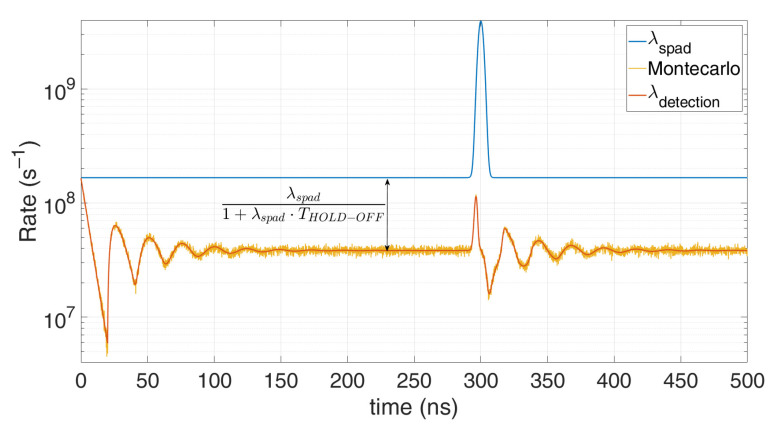
Comparison between a Monte Carlo simulation (yellow) and the proposed model (red), in case of 80 background photons and 20 signal photons within 500 ns simulation time. Note the perfect match; the discrepancy compared to the actual distribution (blue) is due to the SPAD hold-off time.

The result converges to others reported in literature [18,19] and is shown also in Figure 1, which compares this model with a Monte Carlo simulation, in case of 80 background photons within the simulation time (500 ns) and 20 signal photons within a 5 ns FWHM gaussian shape pulse, centred at a 300 ns. All simulations were performed with *T_step_* = 100 ps quantization step and 100,000 repetitions. It can be noticed the heavy pile-up distortion due to the 10 ns hold-off time: the expected peak at 300 ns is shifted toward longer arrival times and gets compressed. Furthermore, in stationary conditions (constant *λ_spad_*) the actual detection rate is lowered by a factor $1+T_{HO}\cdot \lambda_{spad}$. As can be seen, the model perfectly matches simulations since no approximation have been introduced.

### 2.2. Afterpulsing

Now let us add afterpulsing into the model. A SPAD triggering (DET(k)) can have two distinct causes: either a photon detection (PH(k)) or an afterpulsing event (AP(k)). These two events can occur at the same time index k, thus they are not mutually exclusive:

$$\begin{array}{l} P(DET\left(\left(k\right)\right)=P\left(\left(PH\left(k\right)\cup AP\left(k\right)\right)\cap \;\bar{HO}\left(k\right)\right)=\\ =P\left(PH\left(k\right)\cap \;\bar{HO}\left(k\right)\right)+P\left(AP\left(k\right)\cap \;\bar{HO}\left(k\right)\right)-P\left(PH\left(k\right)\cap \;\bar{HO}\left(k\right)\right)\cdot P\left(AP\left(k\right)\cap \;\bar{HO}\left(k\right)\right) \end{array}$$

**Figure 2:**
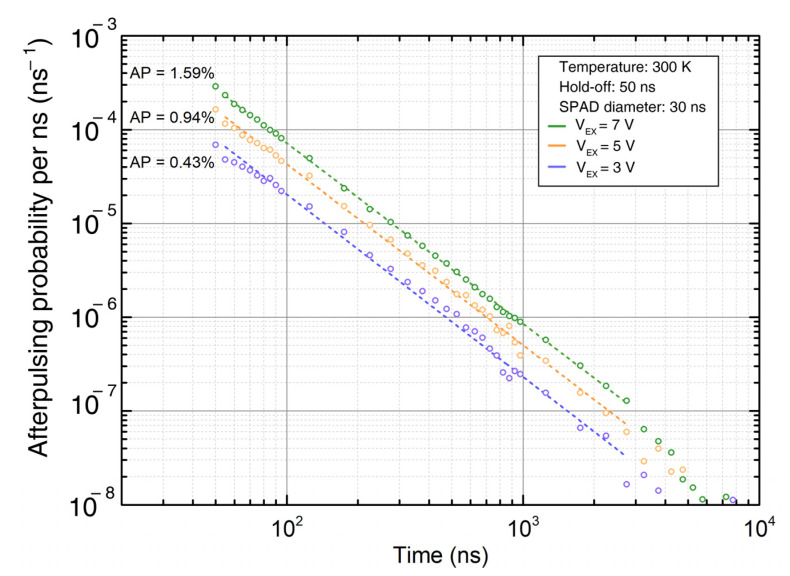
Example of experimental measurements of afterpulsing probability distributions.

The probability of an afterpulsing event AP(k) requires the knowledge of the afterpulsing probability temporal distribution of the detector, i.e., $P\left(AP\left(k\right)|DET_{xy}\left(k-i\right)\right)$, for i ranging from $\Delta_{HO\;}$ to $\Delta_{APmax}$, this last corresponding to the time interval after a photon detection, beyond which the afterpulsing probability is negligible. Such time distribution can be experimentally measured, by observing the time interval between two consecutive SPAD ignitions with no light illumination, after subtracting the dark counts. An example of probability distribution is shown in Figure 2, adapted from [20].

An afterpulsing event at time k can take place only if a detection occurred at instant k−Δ and no detection happened in all following instants:

$$AP\left(k\right)=\cup_{i={\;\Delta }_{HO\;}}^{\Delta_{APmax\;}}DET\left(k-i\right)\cap AP\left(k\right)|DET\left(k-i\right)\cap \cap_{j=k-i+1}^{k-1}\bar{DET}\left(j\right)$$

Since $\cap_{j=k-i+1}^{k-1}\bar{DET}\left(j\right)$ implies $\bar{HO}\left(k\right)$, then $AP\left(k\right)\cap \;\bar{HO}\left(k\right)=AP\left(k\right)$. Hence, being all events $\bar{DET}\left(j\right)$ mutually exclusive, the probability to have an afterpulsing is:

$$P\left(AP\left(k\right)\cap \;\bar{HO}\left(k\right)\right)=\sum_{\;i=\Delta_{HO\;}}^{\Delta_{APmax\;}}P\left(DET\left(k-i\right)\right)\cdot P\left(AP\left(k\right)|DET\left(k-i\right)\right)\cdot P\left(\cap_{j=k-i+1}^{j-1}\bar{DET}\left(j\right)|\;AP\left(k\right)\cap DET\left(k-i\right)\right)$$

Notice that: P(DET(k−i)) is known, being computed in one of the previous times; P(AP(k)|DET(k−i)) is an experimental data (e.g., see Figure 2); and the last term:

$$\cap_{j=k-i+1}^{i-1}\bar{DET}\left(j\right)|\;AP\left(k\right)\cap DET\left(k-i\right)$$

is the probability that no photon has been detected from k−i+1 to k−1. Namely:

- Given DET(k−i), the SPAD is held-off for $k-i+1<j\leq k-i+\Delta_{HO\;}$;
- Being AP(k) the afterpulsing effect, the detection in k−i does not generate afterpulsing within k−i<j<k;
- For every j, given $\cap_{j=k-i+1}^{j-1}\bar{DET}\left(j\right)$, the SPAD is not held-off for $k-i+\Delta_{HO\;}<j<k$.

These considerations result in:

$$\cap_{j=k-i+1}^{k-1}\bar{DET}\left(j\right)|\;AP\left(k\right)\cap DET\left(k-i\right)=e^{\left(-\overset{k-1}{\underset{j=k-i+\Delta_{HOLDOFF\;}+1}{\sum }}\lambda_{spad}\left(j\right)\cdot T_{step}\right)}$$

So, the detection probability in Equation (1) can be written as the sum of two items:

$$\begin{array}{c} P\left(AP\left(k\right)\cap \;\bar{HO}\left(k\right)\right)=\\ \overset{\Delta_{APmax}}{\underset{i=\Delta_{HO\;}}{\sum }}\left(P\left(DET\left(k-i\right)\right)\cdot P\left(AP\left(k\right)|DET\left(k-i\right)\right)\cdot e^{\left(-\overset{k-1}{\underset{k-i+\Delta_{HO}+1}{\sum }}\lambda_{spad}\left(t\right)\cdot T_{step}\right)}\right) \end{array}$$

and: $\left(PH\left(k\right)\cap \;\bar{HO}\left(k\right)\right)=\left(1-e^{-\lambda_{spad}\left(k\right)\cdot \;T_{step}}\right)\cdot \left(1-\overset{k-1}{\underset{j=k-\Delta_{HO\;}}{\sum }}P\left(DET\left(j\right)\right)\right)$ The third term of Equation (1) can be neglected in case of small $T_{step}$.

A stationary regime model can be computed as:

$$\begin{array}{c} \lambda_{detection}=\underset{T_{step}\to 0}{\lim}\frac{P\left(DET\right)}{T_{step}}=\\ =\frac{\lambda_{spad}}{1+\lambda_{spad}\cdot T_{HO}-\int_{T_{HO}}^{t_{APmax}}P\left(AP\left(t\right)|DET\left(t-\Delta t\right)\right)\cdot \exp\left(-\lambda_{spad}\cdot \left(\Delta t-T_{HO}\right)\right)d\Delta t} \end{array}$$

A good approximation is to consider the afterpulsing event right after the end of the hold-off time, i.e., a delta-like afterpulsing probability in time $P\left(AP\left(t\right)|DET\left(t-\Delta t\right)\right)=P_{AP}\cdot \delta \left(t-T_{HO}\right)$ [21], to obtain the detection rate:

$$\lambda_{detection}=\frac{\lambda_{spad}}{1+\lambda_{spad}\cdot T_{HO}-P_{AP}}$$

Such an approximation holds better when afterpulsing probability distribution becomes much shorter than the inverse of the average photon rate, so that $e^{-\lambda_{spad}\cdot \left(\Delta t-T_{HO}\right)}≃1$. In this case, we get the total afterpulsing probability as:

$$\int_{T_{HO}}^{t_{APmax}}P\left(AP\left(t\right)|DET\left(t-\Delta t\right)\right)\cdot e^{-\lambda_{spad}\cdot \left(\Delta t-T_{HO}\right)}d\Delta t≃P_{AP}$$

**Figure 3:**
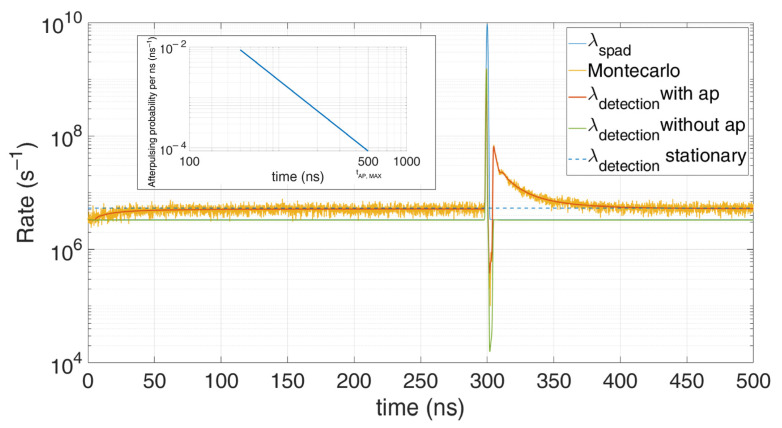
Comparison between Monte Carlo simulations and the model taking into consideration the afterpulsing probability (shown in the inset). Note their perfect match; the distortion compared to the actual expected distribution (blue) is due to the SPAD afterpulsing.

To highlight the afterpulsing effect, in Figure 3 we set low background (1.5 photons) and narrow pulse signal (10 photons in 1 ns FWHM), with 5 ns hold-off time.

### 2.3. Crosstalk

**Figure 4:**
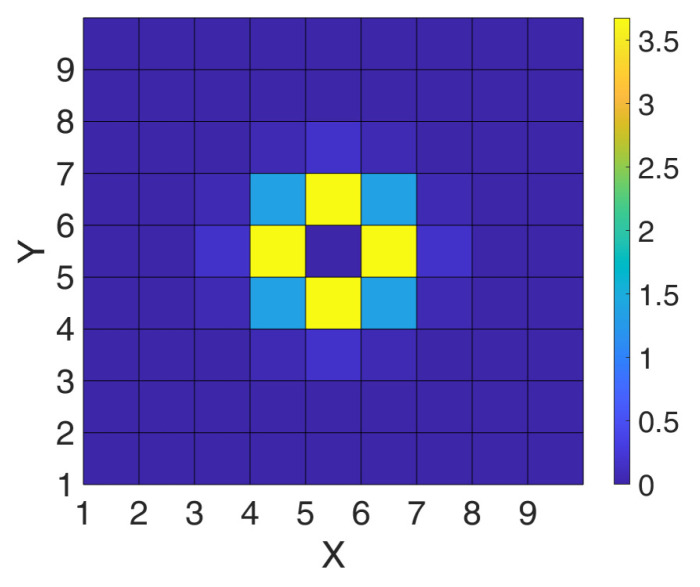
Example of crosstalk probability matrix *P_XT_* in a 9 × 9 SPAD array.

SPAD arrays involve many detectors on the same chip being triggered independently. Such an activity can spuriously trigger neighboring SPADs through optical crosstalk. This phenomenon is defined as the probability to induce a false ignition in a victim SPAD due to a triggering in another aggressor SPAD, after an average delay time T_XT_ linked to the avalanche build-up time. In order to fully characterize crosstalk, a probability matrix needs to be assessed [22], by triggering one specific SPADs (the aggressor) and observing the ignition rates of neighboring victims. Figure 4 shows such a matrix.

The generation event due to crosstalk is defined as *XT_xy_* and depends on all the neighbour SPADs activities:

$$P\left(XT_{xy}\left(k\right)\right)=P\left(\cup_{i,j}XT_{xy\leftarrow i,j}\left(k\right)\;\right)=1-P\left(\cap_{i,j}{\bar{XT}}_{xy\leftarrow i,j}\left(k\right)\right)=1-\prod_{adj}P\left({\bar{XT}}_{xy\leftarrow i,j}\left(k\right)\right)$$

each of them has a certain probability ($P_{XT}$) to generate an avalanche in the xy SPA.

$$P\left({\bar{XT}}_{xy\leftarrow i,j}\left(t\right)\right)=1-P_{XT}{}_{i,j}\cdot P\left(DET_{i,j}\left(t-T_{XT}\right)\right)$$

Now, two events can trigger an avalanche (*PH* and *XT_xy_*) if the SPAD is not in hold-off, so the probability to have a detection at time index k is now:

$$P(DET_{xy}\left(k\right))=P\left(\left(PH_{xy}\left(k\right)\cup XT_{xy}\left(k\right)\right)\cap \;\bar{HO}\left(k\right)\right)$$

$XT_{xy}\left(k\right)$ and $\bar{HO}\left(k\right)$ are not independent, since this second event is related to a possible crosstalk induced from the SPAD to its neighbors (*i*, *j*) that can generate a second crosstalk to the SPAD (*XT_XY_*) in the $\Delta_{HO\;}$. Such a double crosstalk event can be considered negligible for small crosstalk probabilities (***P_XT_*** < 10%), so the two events may be independent.

$$P(DET_{xy}\left(k\right))=P\left(PH_{xy}\left(k\right)\cup XT_{xy}\left(k\right)\right)\cdot P\left(\bar{HO}\left(k\right)\right)$$

and

$$P\left(PH_{xy}\left(k\right)\cup XT_{xy}\left(k\right)\right)=P\left(PH_{xy}\left(k\right)\right)+P\left(XT_{xy}\left(k\right)\right)-P\left(PH_{xy}\left(k\right)\right)\cdot P\left(XT_{xy}\left(k\right)\right)$$

Similar to the afterpulsing model, the last product is negligible for small time steps.

## 3. Modelling of the Acquisition Technique

Many applications rely on time-resolved photon-counting techniques (also known as photon-timing) to attain high timing resolution and precision, from biological applications such as Fluorescence Lifetime Imaging (FLIM) [23], near-infrared spectroscopy [24], to VLSI chip inspection [25], to looking-around the corner vision [[] to LiDAR [26]. In this section, we investigate two main photon-timing techniques: classical Time-Correlated Single Photon Counting (TCSPC), usually being single-hit and with SPAD reset synchronous with the excitation laser pulse; and multi-hit Time-of-Flight (TOF) measurements, with free-running SPAD and multiple detections of photons within the frame time. The former is single-hit because it registers the timing information of just the first photon and, by creating a histogram, it reconstructs the incoming light waveform in photon starving application, when at the most one photon per laser shot is expected. Instead, the latter technique can be exploited at high counting rates, when more than one photon is received per laser excitation. Ideally, all photons should be acquired, though this is impossible due to the SPAD dead-time and the dead-time of the timing electronics (e.g., the TDC conversion time) and other resources limitations (e.g., on-chip memory availability and readout speed). The following subsections model some techniques copying those effects.

### 3.1. Single-Hit Detection

TCSPC is the key technique to acquire very faint signals down to the picosecond time scale; however, particular care on the count rate must be taken. Since the technique relies on the detection and timestamping of the first photon’s timing information, this gives rise to a strong “pile-up” distortion when more than one photon reaches the detector, but only the first one is recorded. This effect is negligible for count rates lower than 5% of the laser frequency [27,28], i.e., when the probability to have more than one photon is less than 0.1% for each laser excitation, as predicted by Poisson statistics. In fact, the probability that a photon be detected at a given time, if no photon has been detected yet, is:

$$P\left(PH\left(t\right)\right)=1-e^{-\lambda_{spad}\left(t\right)\cdot \;T_{step}}$$

In case of single-hit technique, the detection at time *t* is possible only if an avalanche is generated *and* no photon has been revealed previously since the start of the observation time (namely, the last laser excitation). Hence:

$$P(\cap_{k=0}^{t}\;\bar{DET\left(k\right)})=e^{-\overset{t}{\underset{k=0}{\sum }}\lambda_{spad}\left(k\right)\cdot \;T_{step}}$$

These two events being independent [29], we get:

$$P\left(DET\left(t\right)\right)=e^{-\overset{t}{\underset{k=0}{\sum }}\lambda_{spad}\left(k\right)\cdot \;T_{step}}\cdot \left(1-e^{-\lambda_{spad}\left(t\right)\cdot \;T_{step}}\right)$$

The same result [30] can be achieved by exploiting the recursive expression as in Equation (1), by prolonging the hold-off to the whole observation time before time index *k*:

$$P(\cap_{k=0}^{t}\;\bar{DET\left(k\right)})=P(1-\cup_{j=0}^{k-1}DET_{\;}\left(j\right))=1-\sum_{j=k-\Delta_{HO\;}}^{k-1}P\left(DET\left(j\right)\right)$$

So, to get:

(2) $$P\left(DET\left(k\right)\right)=\left(1-e^{-\lambda_{spad}\left(k\right)\cdot \;T_{step}}\right)\cdot \left(1-\sum_{j=0}^{k-1}P\left(DET\left(j\right)\right)\right)$$

**Figure 5:**
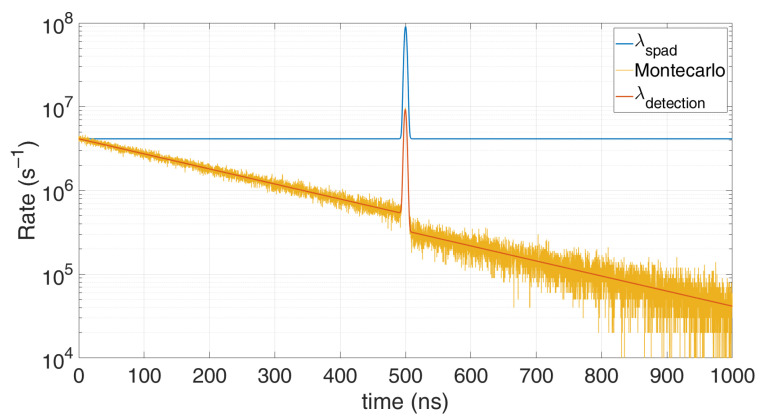
Comparison of the TCSPC model (red) with the Monte Carlo simulation (yellow), in the case of 0.5 signal photons and 4 noise photons on average within the 1 µs observation time. Note their perfect match; the decaying trend compared to the actual expected distribution (blue) is due to pile-up caused by the single-hit acquisition.

Figure 5 shows how this model perfectly matches the Monte Carlo simulation: an average of 0.5 signal photons and four noise photons are considered within the 1 µs observation time. The typical exponential decay due to pile-up distortion is clearly visible.

### 3.2. Multi-Hit Detection

Most TOF applications cannot trust on single photon regime, for example due to the presence of high-level background light, which can represent a huge source of noise, even up to 100 klx in many automotive environments. While luminance refers to the amount of light that passes through, emits from, or reflects off an object and falls within a given solid angle (and is measured in candela per square meter, cd/m^2^), illuminance refers to the total amount of light incident onto a given surface, per unit area, and is measured in lux (lx, i.e., lumens per squared meter) and is wavelength-weighted to correlate with human brightness perception. Illuminance is often called brightness, but the latter is meant just for nonquantitative references to physiological sensations and perceptions of light. Actually, in the LiDAR field, background light is often quoted in terms of lux, both by companies and by literature. Other typical requirements in TOF LiDAR are high laser pulse energy and high dynamic range, needed to reveal object from few meters up to hundreds of meters.

Therefore, all these applications cannot rely on the detection of just the first returning photon after each laser shot, such as in classical single-hit TCPSC techniques, because measurements would show a huge pile-up distortion (as shown in Figure 5). The ideal solution would be to have no dead-time due to either the detector (e.g., the SPAD hold-off time) or the timing electronics (e.g., the TDC conversion time), and the possibility to record the highest number of TOF acquisitions (ideally all photons’ TOFs) within the frame-time (e.g., the maximum distance full-scale range) after each laser shot. Of course, this is not possible, due to SPAD hold-off time (usually some ns), TDC dead-time (from few ns to some µs, depending on the architecture), limited readout bandwidth (above all with laser repetition exceeding some MHz) and limited storage availability. Short hold-off SPADs, fast re-triggerable TDCs, and fast timing architectures are being studied to achieve shorter and shorter dead-times. Furthermore, histogram generation and accumulation and TOF centroid evaluation and readout are being implemented on-chip to avoid the bottleneck of chip’s readout bandwidth and off-chip data storage and processing. Some studies in literature try to model the combined dead-time effect [31]; however, that is feasible when considering a specific architecture, e.g., a SPAD with a TDC [32]. Instead, for more complex architectures that modelling becomes very difficult to implement. In the following, we develop a statistical model able to describe the timing electronics dead-time (T_DEAD_) and also the limited number of TOF data to be store in a frame for the case of a multi-hit architecture.

**Figure 6:**
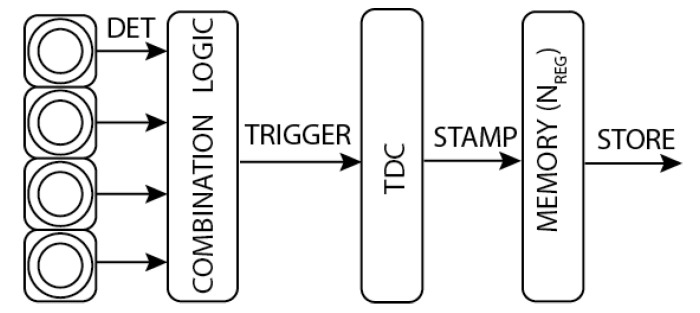
Typical photon-timing chain, from photon detection to Time-of-Flight storage.

Figure 6 shows a generic detection architecture employing either a single SPAD or a combination of SPADs (such as in a SiPM) to provide one output event a time. The detector provides a defined event rate (i.e., single photons, coincidence photons, or logic-OR combination among SPADs, etc.) that trigger one single TDC, resulting in the TOF time-stamp to be stored into a register. As shown in Figure 6, a time-stamp is generated when a TRIGGER event triggers the timing electronics, which was quiescent for at least the previous T_DEAD_ time.

In order to simplify the model, we introduce an approximation: the trigger probability is considered to be independent of the previous time instant, since the analytical dependency on what happened in the previous T_DEAD_ is not straightforward to compute. Therefore, similarly to the model with just hold-off (see Par. 2), a TDC conversion (STAMP event) occurs whether a trigger event takes place and no conversion happened in the last TDC dead-time:

(3) $$\begin{array}{ll} P\left(STAMP\left(k\right)\right) & =P\left(TRIGGER\left(k\right)\cap \;\overset{k-1}{\underset{j=k-\Delta_{DEAD\;}}{\cap }}\;\bar{STAMP\left(j\right)}\right)\\ & =P\left(TRIGGER\left(k\right)\right)\cdot P\left(\overset{k-1}{\underset{j=k-\Delta_{DEAD}}{\cap }}\;\bar{STAMP\left(j\right)}\;|\;TRIGGER\left(k\right)\right)\\ & ≃P\left(TRIGGER\left(k\right)\right)\cdot \left(1-\overset{k-1}{\underset{j=k-\Delta_{DEAD\;}}{\sum }}P\left(STAMP\left(j\right)\right)\right) \end{array}$$

**Figure 7:**
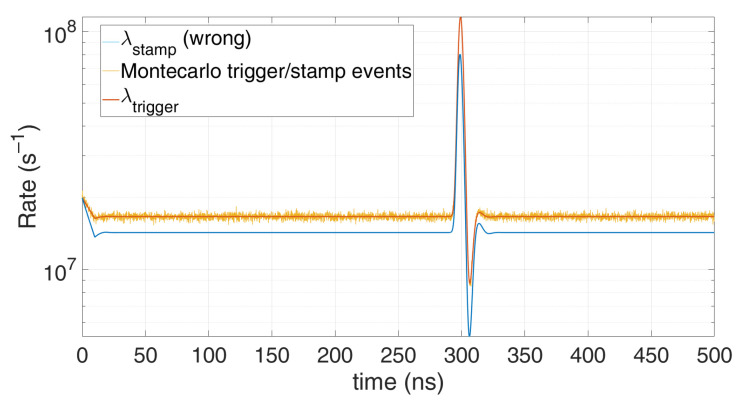
Comparison between Monte Carlo simulation and model with timing electronics dead-time, single SPAD, T_HO_ ≥ T_DEAD_ = 10 ns (worst case, when the approximation does not hold, so Equation (1) should be used instead of Equation (3)), with 1 signal photon and 10 noise photons on average per repetition.

**Figure 8:**
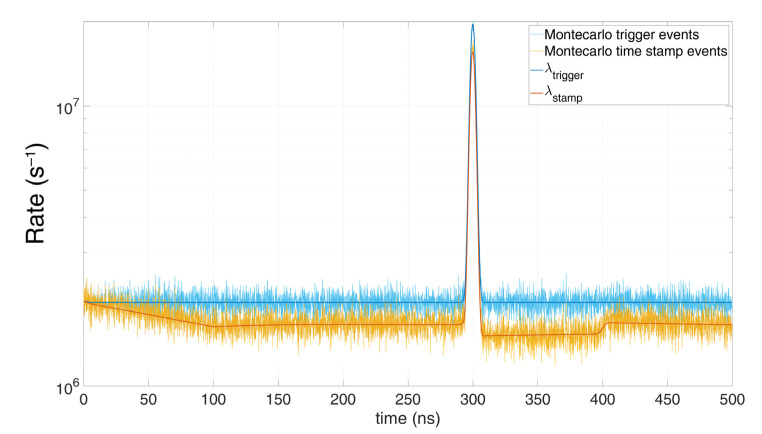
Comparison between Monte Carlo simulation and model with timing electronics dead-time, single SPAD, T_HO_ > T_DEAD_, with 0.1 signal photon and 1 noise photon on average per repetition (T_HO_ = 5 ns, T_DEAD_ = 100 ns).

For single SPAD architectures (i.e., no combination logic), if the detector dead-time is dominant, i.e., $T_{HO}\;\geq T_{DEAD}$, we have:

$$P\left(\cap_{j=k-\Delta_{DEAD\;}}^{k-1}\;\bar{STAMP\left(j\right)}\;|\;TRIGGER\left(k\right)\right)=1$$

and P(STAMP(k))=P(TRIGGER(k))=P(DET(k)), as in Equation (1). In this case, the first model described by Equation (1) fits perfectly, since only one time stamp can occur after one detection. However due to the approximation, the model in Equation (3) gives the error shown in Figure 7. Conversely, in case the SPAD hold-off time is negligible compared to the TDC dead-time, the dependency of TRIGGER event with the previous instants is very weak, so the approximation holds better, as shown in Figure 8.

Now, let us consider the limitations in reading out and storing the TOF data: one measure can be stored (STORE event) when a time-stamp occurs (STAMP event) and the memory is not saturated. At first, we consider a trigger event corresponding to a time-stamp, i.e., no TDC dead-time for now, then:

$$P\left(STORE(k\right)=P\left(STAMP\left(k\right)\cap \;\bar{SAT}\left(k\right)\right)$$

A measurements saturate (SAT(k)=1) if the number of time stamps before the i-th bin is higher or equal to the available number of storage registers $N_{REG}$:

$$P\left(\bar{SAT}\left(k\right)\right)=P\left(N_{STAMP}\left(k-1\right)<N_{REG}\right)$$

Since P(STAMP) depends on the photon rate, it is not constant over time. Thus, the exact computation of $P\left(\bar{SAT}\left(k\right)\right)$ would require the computation of all possible combinations with $N_{STAMP}\left(k-1\right)<N_{REG}$. Within a $T_{obs}$ observation time, in the worst case there are $\left(\begin{array}{c} N_{TOTAL\;BIN}\;\\ N_{REG} \end{array}\right)$ possible combinations, whose analytic computation is not feasible. Moreover, it would be also necessary to know the dependency between different STAMP(k) events. In order to simplify the analysis, we made the following assumptions:

- All STAMP(k) events are independent;
- Being P(STAMP(k)) almost constant within the whole observation period apart from the peak duration, the probability distribution in the previous k−1 windows ($N_{STAMP}\left(k-1\right)$) is considered a binomial distribution with a number of trials n(k)=k−1 and a probability per trial equal to the average probability: $p\left(k\right)=\frac{\sum_{j\in \left[1,k-1\right]}P\left(STAMP\left(j\right)\;\right)\;}{n}$.

The binomial probability is defined as:

$$B\left(n,i,p\right)=\left(\begin{array}{c} n\left(k\right)\\ i \end{array}\right)\cdot p{\left(k\right)}^{i}\cdot {\left(1-p\left(k\right)\right)}^{n\left(k\right)-i}$$

By applying the assumptions, $P\left(\bar{SAT}\left(k\right)\right)$ and P(STORE(k)) can be computed as:

(4) $$\begin{array}{l} P\left(\bar{SAT}\left(k\right)\right)=P\left(N_{coinc}\left(k-1\right)<N_{sat}\right)=\sum_{i=0}^{N_{sat}-1}B\left(n\left(k\right),i,p\left(k\right)\right)\\ P\left(STORE\left(k\right)\right)=P\left(STAMP\left(k\right)\;\cap \;\bar{SAT}\left(k\right)\right)=P\left(STAMP\left(k\right)\right)\cdot P\left(\;\bar{SAT}\left(k\right)\right) \end{array}$$

**Figure 9:**
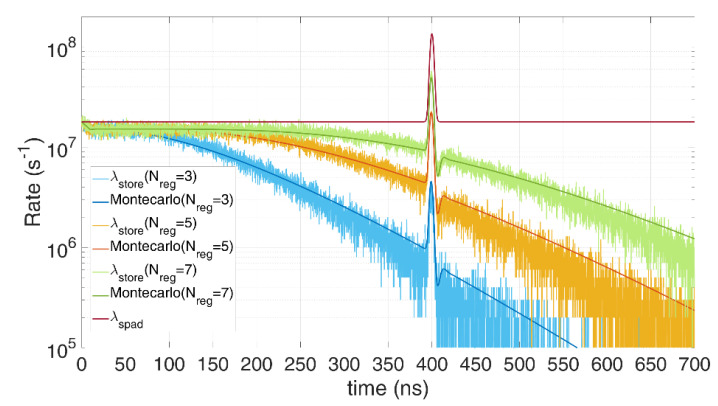
Comparison between Monte Carlo simulations and model in case of register limitations (3, 5, or 7 maximum TOF storable values), single SPAD, with 0.6 signal photons and 12 noise photons on average per repetition (T_HO_ = 10 ns). Note the perfect matches; the distortion compared to the actual expected distribution (blue and red top lines) is due to the limited available resources.

Figure 9 shows the pile-up distortion due to the limited number of registers, the first measurements (at shorter time) have higher probability to be stored since storage registers are still empty. The assumptions made to simplify the model can still provide a good approximation. In the following, in order to avoid such a heavy distortion, we investigate three different solutions, namely, photon coincidence, time gating, and multi-TDCs.

### 3.3. Coincidence Detection

In order to reduce the impact of the detector dead-time and to provide a sort of background suppression, one possibility is to exploit multi-SPAD pixel architectures and photon coincidence [30]. Even if background photons trigger a large number of SPADs within the sensor at a certain rate, these events are usually random and uncorrelated; instead, signal photons arrive at a precise moment in time, the TOF of the laser pulse, so they are coincident within the laser pulse width duration. Therefore, as soon as a defined number of SPADs fire in such a coincidence time window, the coincidence logic triggers the timing electronics to compute the signal TOF, while being almost insensitive to background. Hence, this solution reduces the probability to saturate measurements and resources, because the timing electronics is triggered only in case of useful signal photons.

Let us now consider one single pixel with one TDC and an array of m × n SPADs, acting just as a single active area (unlike an x and y imager). The SPADs outputs are combined into an AND-like logic gate, which provides a pulse to the TDC only if at least *i* ≥ *N_t_* SPADs trigger concurrently. Here, we compute the probability P(COINC(j)) that N_t_ photons trigger the TDC in a coincidence time window j.

At first, we neglect any crosstalk so all SPAD events can be considered independent of each other. Two different coincidence approaches are investigated: fixed-window and moving window. The former is used in synchronous systems, where the sum of SPADs contributions is observed at the end of each coincidence time window, by checking if the N_t_ threshold is exceeded. Instead, the latter is implemented with a suitable asynchronous coincidence electronics.

#### 3.3.1. Fixed Coincidence Window

With a uniform photon distribution among the m × n SPADs in the same pixel, the probability that a photon hits one specific SPAD (of coordinated *x*, *y*) is given by Equation (1). Since detection events in a SPAD are mutually exclusive (considering $T_{window}<T_{HO}$), the probability of having a detection in a time window j (whose width index is Δ*_window_* = *T_window_*/*T_step_*) is given by:

$$P\left(DET_{xy,\Delta_{window}}\left(j\right)\right)=P\left(\cup_{k\;\in \;\Delta_{window}}DET_{xy}\left(k\right)\right)=\sum_{k\;\in \;\Delta_{window}}P\left(DET_{xy}\left(k\right)\right)\;$$

A coincidence event $COINC_{N_{t}}\left(j\right)$ occurs when at least $N_{t}$ detections arise in the same coincidence window ($DET_{xy,T_{window}}\left(j\right)$). Indicating with $comb_{}$ one of the possible $N_{comb}$ combinations to have $N_{DET}$ SPADs triggered among $N_{SPAD}$ available ones, the probability of having a coincidence in the window *j* is:

$$P\left(COINC_{N_{t}}\left(j\right)\right)=P\left(\cup_{N_{DET}=N_{t}}^{N_{spad}}\left(\cup_{comb_{x}=1}^{N_{comb}}\left(\cap_{spad_{index}=1}^{N_{spad}}EVENT\left(spad_{index}\right)\;\right)\right)\right)$$

where $N_{comb}=\left(\begin{array}{c} N_{spad}\\ N_{DET} \end{array}\right)$ and

$$EVENT\left(spad_{index}\right)=\{\begin{array}{c} DET_{spad_{index},\Delta_{window}}\left(j\right)\;\mathrm{if}\;spad_{index}\in combination\;\\ \bar{DET_{spad_{index},\Delta_{window}}}\left(j\right)\;\mathrm{if}\;spad_{index}\notin combination \end{array}$$

In the case under exam, the probability of a detection event can be considered constant among different SPADs, thus the expression can be simplified to:

$$P\left(COINC_{N_{t}}\left(j\right)\right)=\sum_{N_{DET}=N_{t}}^{N_{spad}}B\left(N_{spad},\;N_{DET},P\left(DET_{Twindow}\left(j\right)\right)\;\right)$$

**Figure 10:**
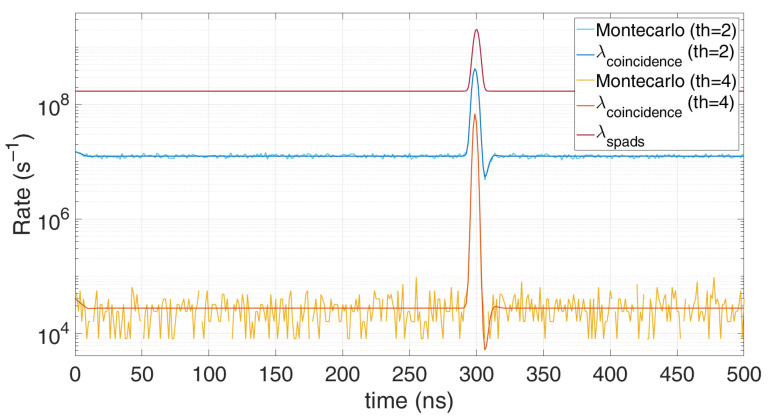
Comparison between Monte Carlo simulation and model for fixed-window photon coincidence, at two photon thresholds (*N_t_* = 2 or 4), with 10 signal photons and 100 noise photons on average per repetition (*T_HO_* = 10 ns). Note the effective rejection of the background baseline by a factor of about 10,000 in case of TOF measured only when at least 4 SPADs trigger concurrently.

Figure 10 shows the clear benefit of photon coincidence in a group of 16 SPADs: the probability to collect background events compared to the signal gets significantly lower when setting a threshold *N_t_* of 2 and 4 photons, by a factor 10 and 10,000, respectively.

#### 3.3.2. Moving Coincidence Window

The second approach exploits a sliding coincidence window and can be implemented in asynchronous systems. Conversely to the fixed coincident case, which requires to sample the counting electronics at every time window step, this method can be implemented by shaping the SPAD digital pulse with a width equal to the desired coincidence time window interval. Then, all these digital pulses are summed in intensity and a coincidence occurs every time the total intensity crosses a given analog threshold, proportional to the desired photon threshold *N_t_*.

The probability to have a coincidence at time index *k* is defined as the probability to have *i* detections in the previous coincidence window (indexes from *k* − Δ*_window_* to *k* − 1) and the probability to have *N_t_* − *i* detections in the current time index *k*:

$$P\left(COINC\left(k\right)\right)=\sum_{i=0}^{N_{t}-1}P\left(N_{DET}\left(k-\Delta_{window},k-1\right)=i\right)\cdot P(N_{DET}\left(k\right)\;\geq N_{t}-i\;|\;N_{DET}\left(k-\Delta_{window},k-1\right)=i\;)$$

Such probabilities follow the binomial distribution:

$$P\left(COINC\left(k\right)\right)=\sum_{i=0}^{N_{t}-1}\left(B\left(N_{spad},\;i,\sum_{j=k-\Delta_{window}-1}^{k-1}P\left(DET\left(j\right)\right)\right)\cdot \cdot \sum_{m=Nt-i}^{N_{spad}}B\left(N_{spad}-i,\;m,P\left(DET\left(k\right)\right)\right)\right)$$

If *T_step_* is very short, the probability to have more than one detection among the *N_spad_* at time index *k* is almost nil; therefore, in such a case the coincidence probability can be rewritten as the probability that one photon is detected by the group of SPAD at time k and *N_t_* − 1 have been detected within the $\Delta_{window}-1$ previous time indexes. We get:

$$P\left(COINC\left(k\right)\right)=B\left(N_{spad},\;\sum_{j=k-\Delta_{window}-1}^{k-1}P\left(DET\left(j\right)\right),N_{t}-1\right)\cdot {\left(1-\left(1-P\left(DET\left(j\right)\right)\right)\right)}^{N_{spad}-\left(N_{t}-1\right)}$$

**Figure 11:**
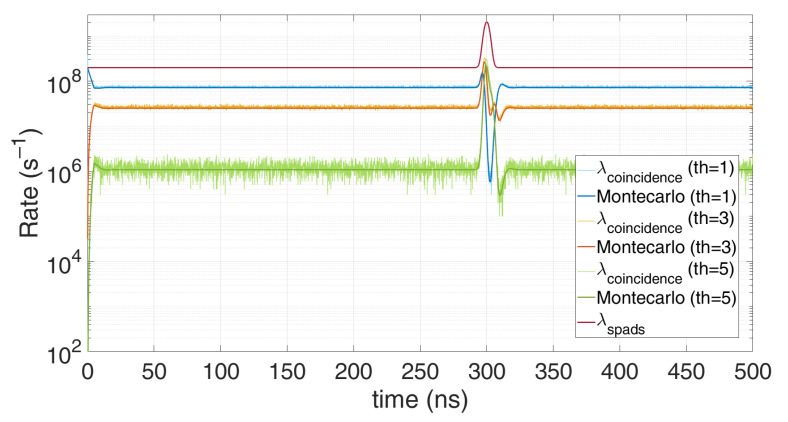
Comparison between Monte Carlo simulations and model for a moving photon coincidence window, at different thresholds, for 10 signal photons and 100 background photons on average per repetition (*T_HO_* = 10 ns).

As for fixed coincidence window, the higher the threshold, the better the noise filtering, as shown in Figure 11. A high coincidence threshold lets just fewer measures to trigger the TDC and occupy a memory cell. However, too high threshold causes the probability to detect signal photons to lower as well.

**Figure 12:**
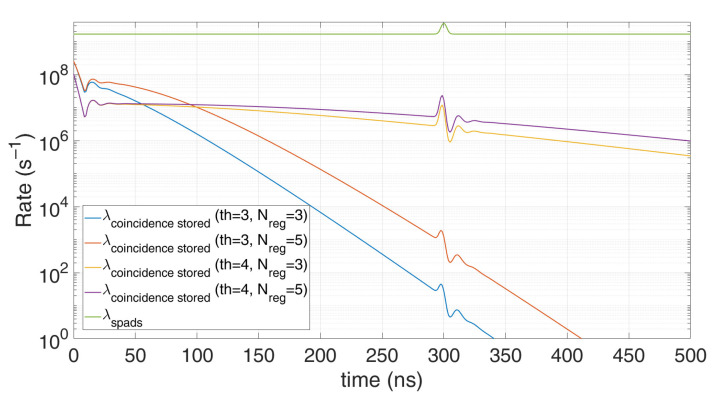
Example of modelling a limited number of TOF registers per laser pulse, with different photon threshold and number of registers, for 0.6 signal photons and 62 background photons on average per repetition (*T_HO_* = 10 ns). Note how distortion increases for lower coincidence threshold and for lower TOF register availability per laser pulse repetition.

Figure 12 shows an example of coincidence photons acquisitions when background (62 photons on average per laser pulse repetition) overwhelms signal (only 0.6 photons on average per repetition). Only a sufficiently high photon coincidence threshold (e.g., 4 instead of 3 in the figure) and the availability of a sufficient number of registers to store the TOFs in each laser shot (e.g., 5 instead of 3 in the figure) allows to acquire a low-distorted histogram (line in purple color) with high signal level, with strong background rejection (about a factor 100) whilst limited signal loss.

### 3.4. Time-Gated Detection

The possibility to turn on the detector within a defined enabling time window is a convenient way to avoid detecting and time-stamping useless photons. Such time-gating approach requires the knowledge of the time interval where the signal is expected, providing a GATE control signal to the detection electronics. In another approach, time gating can be exploited to perform repetitive measurements within a given gate window and then sliding it across the desired full-scale range of the acquisition.

The new model takes into account the saturation to *N_reg_* measurements (see previous Section 3.2), to see the time-gate effect. The probability of not saturate the memory ($P\left(\bar{SAT}\left(k\right)\right)$) is computed as in Equation (4). Let us define $GATE_{start}$ and $GATE_{stop}$ the time indexes of the starting (gate-on) and the ending (gate-off) time of the GATE, respectively.

The distribution describing $N_{TRIGGER}\left(k-1\right)$ events, i.e., photon coincidences measured before the window i, for $i\in \left[GATE_{start},GATE_{stop}\right]$ becomes:

$$P\left(\bar{SAT}\left(k\right)\right)=\overset{N_{sat}-1}{\underset{N_{coinc}=0}{\sum }}\left(\begin{array}{c} n\left(k\right)\\ N_{TRIGGER} \end{array}\right)\;p{\left(i\right)}^{N_{TRIGGER}}\cdot {\left(1-p\left(k\right)\right)}^{n\left(k\right)-N_{TRIGGER}}\cdot P\left(MEAS\left(GATE_{start}\leq k\leq GATE_{stop}\right)\right)=P\left(COINC_{N_{t}}\left(GATE_{start}\leq k\leq GATE_{stop}\right)\right)\cdot P\left(\;\bar{SAT}\left(k\right)\right)\cdot P\left(MEAS(k\langle GATE_{start}\;||\;k\rangle GATE_{stop}\right)=0$$

where $n\left(k\right)=k-GATE_{start}$ and $p\left(k\right)=\frac{\sum_{j\in \left[GATE_{start},k-1\right]}P\left(COINC_{N_{t}}\left(j\right)\;\right)\;}{n}$.

**Figure 13:**
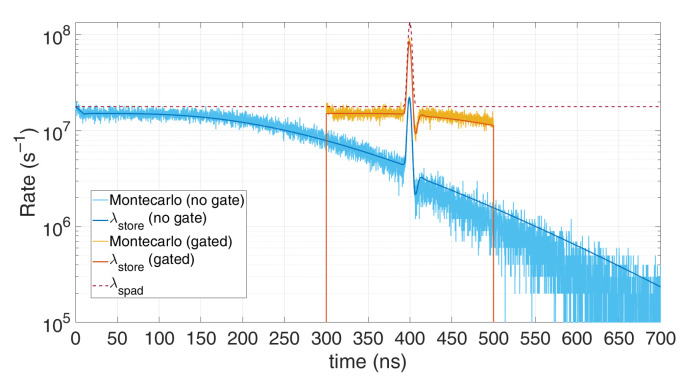
Comparison between Monte Carlo simulation and modelling for time-gated detection, in case of 7 registers and a single SPAD, with 0.6 signal photons and 12 background photons on average per repetition (T_HO_ = 10 ns). Note how time gating reduces distortion, since TOF memory registers are not wasted for background photons before the gate window.

Figure 13 clearly shows the measurements improvement with time gating: much less distortion is achieved, since no memory storage gets wasted for background photons.

### 3.5. Multi-TDCs Architectures

The last method relies on the possibility to measure the TOF through more TDCs per detector, in order to reduce the impact of the timing electronics dead-time, by routing the detector to the first available TDC [31]. A time stamp is possible if an event triggers the TDC and the number of TDC is not saturated ($\bar{TDCSAT}$). Again, for simplicity, the trigger probability is considered independent of what happened before, hence:

$$P\left(STAMP\left(k\right)\right)=P\left(TRIGGER\left(k\right)\;\cap \;\bar{TDCSAT}\left(k\right)\right)=P(TRIGGER\left(k\right)\cdot P\left(\bar{TDCSAT\left(k\right)}\right)$$

Similarly to the computation of P(SAT) in Equation (4), the probability not to saturate the available number of TDCs (*N_TDC_*) can be defined as:

$$P\left(\bar{TDCSAT}\left(k\right)\right)=P\left(N_{busy\;TDC\;}\left(k-1\right)<N_{TDC}\right)=\sum_{i=0}^{N_{TDC-1}}B\left(\Delta_{dead},\;i,p\left(k\right)\right)$$

with

$$p\left(k\right)=\frac{\sum_{j\in \left[k-\Delta_{dead},\;k-1\right]}P\left(STAMP\left(j\right)\;\right)\;}{\Delta_{dead}}$$

**Figure 14:**
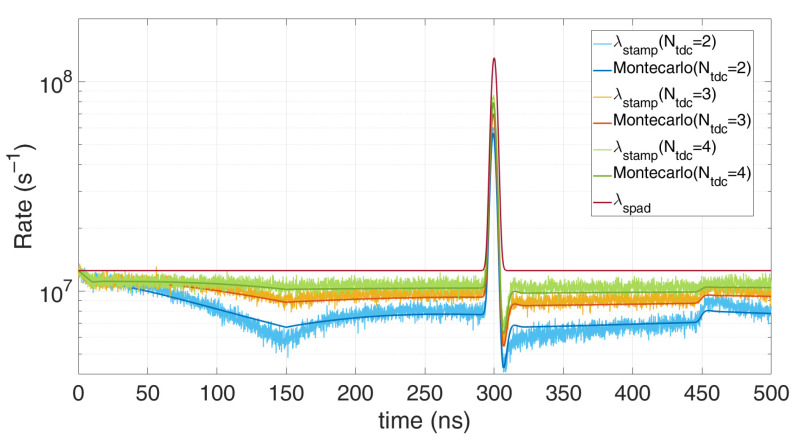
Comparison of Monte Carlo simulations and modelling for *N_tdc_* multi-TDCs and single SPAD, with 0.6 signal photons and 6 background photons on average per repetition (*T_HO_* = 10 ns, T_DEAD_ = 150 ns).

Of course, the higher the number of available TDC, the closest the measurement gets to the actual incoming rate. Figure 14 shows the excellent matching of the model with the Monte Carlo simulations, at different number of available multi-TDCs. The minor mismatches are due to the implemented simplification. It can be noticed the TDC dead-time effect, introducing a first pile-up until 150 ns and a second pile-up right after the laser pulse.

## 4. Distortion Correction

Up to now, we presented a detailed analytical modelling of different detector configuration (Section 2) and electronic acquisitions (Section 3). Now, we show how the developed statistical approach can be profitably used to predict the effect of non-idealities of SPAD-based detection systems. Furthermore, in some specific use-cases, the modelling can be exploited to correct the experimentally acquired histogram, featuring distortions introduced by the sensor, so as to extract a better estimation of the actual signal of interest. In the following subsections, we consider two typical situations.

### 4.1. TCSPC Correction

Time-Correlated Single Photon Counting (TCSPC) is usually applied with extremely faint photon sources, which provide less than one photon on average. Various works in literature deal with statistical ways to correct distorted TCSPC histograms in case of few photons, for example [27,33].

We propose another correction by starting from the modelling presented in Equation (2) and by computing an estimation of *λ_spad_*. We start from the detection probability histogram P(DET(k)), given by the measured histogram divided by the number of pulses. An estimation ${\hat{\lambda }}_{spad}$ of the detection probability can be derived from the measured histogram, normalized to the number of pulses, by means of [34]:

$${\hat{\lambda }}_{spad}\left(k\right)=-\ln\left(1-\frac{h\left(k\right)}{N_{pulses}-\sum_{j=0}^{k-1}h\left(j\right)}\right)$$

Notice the correction (amplification) of the measured data, due to the denominator terms, so that the higher *N_pulses_* gets, the better and less noisy the correction becomes, because the acquired histogram gets less noisy.

By implementing this correction, it is possible to break the TCPSC rule of thumb stating that the incoming rate should be less than 5% of the repetition frequency in order to avoid excessive pile-up distortion due to the first photon masking all subsequent ones [29]. Unfortunately, such a constraint drastically increases the measurement time, since no photon is detected for at least 95% of the laser shot repetitions.

Concerning the model, if the incoming flux is too high, the probability to have no photon detections in a repetition tends to zero; therefore, for a given number of repetitions, $N_{pulses}-\overset{k-1}{\underset{j=0}{\sum }}h\left(j\right)$ vanishes and ${\hat{\lambda }}_{spad}$ diverges. Such a condition must be avoided, by limiting the incoming flux and by increasing the probability to have some repetitions with no photon arrivals, namely:

$$N_{pulses}\cdot p\left(0\;photons\right)=N_{pulses}\cdot exp\left(\sum_{k=\left[1,\;N_{tot\;bins}\right]}\lambda_{spad}\left(k\right)\cdot \Delta t_{bin}\right)$$

Thereby, in order to have more than *k* empty repetitions we must have $p_{\left(0\;photons\right)}>\frac{k}{N_{pulses\;}}$.

**Figure 15:**
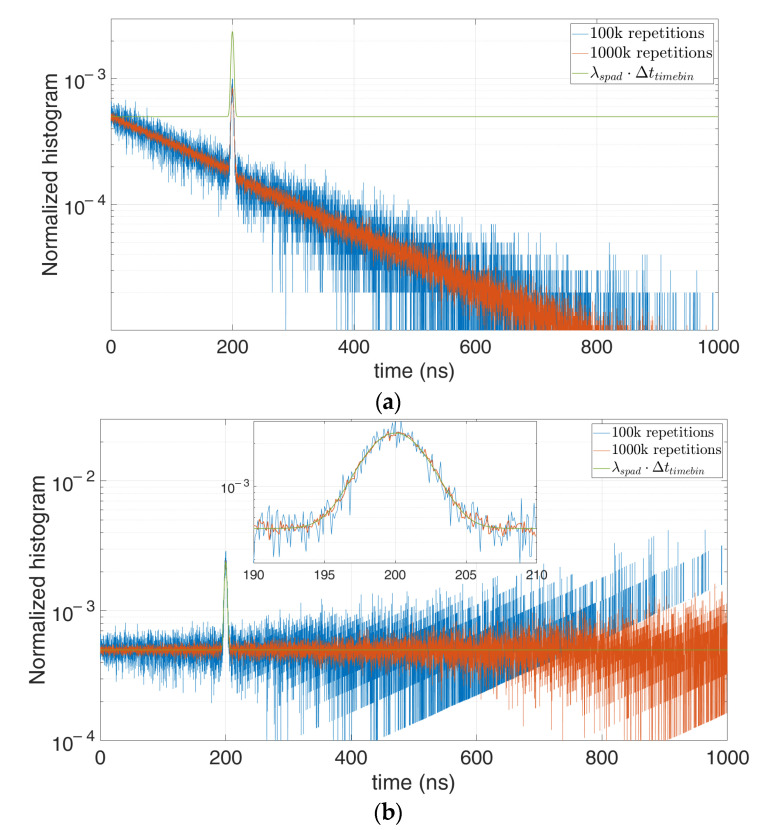
Distorted histogram in a single-photon TCSPC setup (**a**) and corrected estimation of the actual original signal (**b**), in case of 0.1 signal photon and 1 background photon on average per repetition, at 100,000 (blue) and 1,000,000 (red) repetitions.

We performed different simulations with different number of repetitions and average number of signal and background photons. For example, let us consider 0.1 signal photon and 1 background photon on average per laser shot repetition, so as to get $p_{\left(0\;photons\right)}=35\%$. Figure 15 shows that the higher the number of repetitions becomes, the less noisy the measured histogram gets and the better the correction results. 

Indeed, the number of k empty repetitions increases (from 35,000 to 350,000 in Figure 15, for the case with 100,000 and 1,000,000 repetitions, respectively) and the error at higher time bins decreases. We can conclude that thanks to the correction a lower p(0 photons) can be allowed, giving the possibility to perform a measure with either higher photon flux or observation time (affording way less than 95% of p(0 photons)).

### 4.2. Multi-Photon Correction

The same approach can be applied to free-running applications with more photon detections within the observation time. The corresponding correction method can be employed both in high background applications and whenever a distortion due to fixed dead-time becomes severe. An example is a detection system employing a single SPAD with a hold-off time being the main limitation, or an architecture with a generic event that could trigger the TDC (a combination of SPADs) and the assumptions made in Equation (3) (trigger event independent of previous instants). In these cases, a good estimation is:

$${\hat{\lambda }}_{spad}\left(k\right)=-\ln\left(1-\frac{h\left(k\right)}{N_{pulses}-\sum_{j=k-\Delta_{\mathrm{dead}}\;}^{k-1}h\left(j\right)}\right)$$

**Figure 16:**
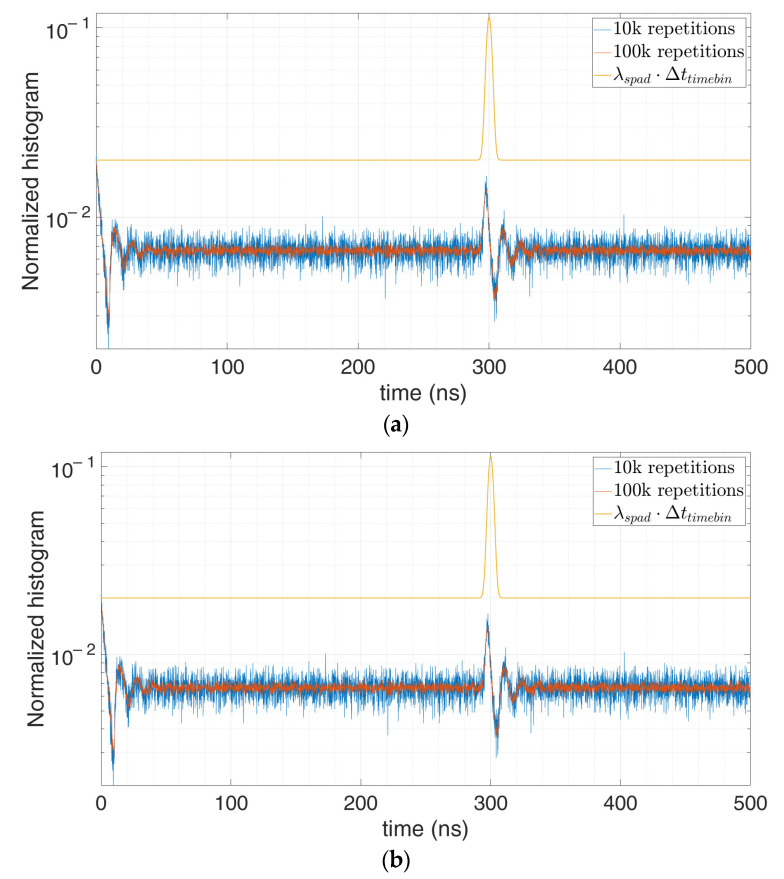
Distorted histogram in a multi-photon setup (**a**) and corrected estimation of the actual original signal (**b**) for single SPAD and single TDC architecture, in case of 5 signal photons and 100 background photons on average per repetition (T_HO_ = 10 ns) at 100,000 (blue) and 1,000,000 (red) repetitions.

Figure 16b shows the correction of the distorted Figure 16a in case of a single SPAD feeding a single TDC, whose dead time is shorter than the SPAD hold-off time.

**Figure 17:**
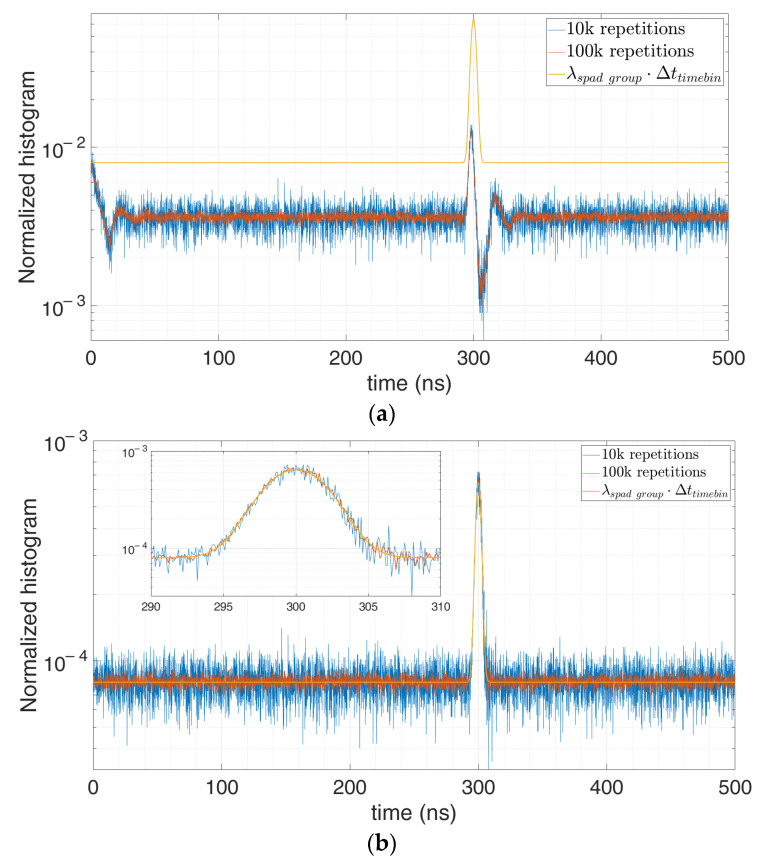
Distorted histogram in a multi-photon setup (**a**) and corrected estimation of the actual original signal (**b**) for 100 SPADs in a global OR combination, driving one TDC, with 3 signal photons and 40 background photons on average per repetition (on the cluster) (T_HO_ = 10 ns, T_DEAD_ = 15 ns), at 10,000 (blue) and 100,000 (red) repetitions.

However, the same model can be used also when the timing electronics dead-time is longer and is the main limitation, by exploiting Equation (3). The more the trigger event is independent of previous instants, the more reliable the estimation becomes. An example could be when more SPADs feeding a combination logic drive one single TDC. Figure 17 shows the results in case of 100 SPADs in global OR triggering one TDC. In these cases, the more the SPADs the lower the dependency of the trigger event (OR among the SPADs).

Notice that any other model discussed in this work does not offer the advantage to be reversed in order to estimate the actual original signal. However, all of them can be implemented in iterative algorithms, starting from an estimation of the external conditions, computing the histogram through the proposed modelling, and then reducing the error between initial guess and computed histogram, by updating parameters such as background, reflectivity, and distance of the object at every cycle. Once the model converges, the *λ_SPAD_* is eventually estimated. Such methodology can be used in photon coincidence application, taking advantage of the applicable model among those discussed in the previous sections, namely, fixed and moving coincidence, gating and multi-TDC architectures.

## 5. Conclusions

SPAD based system often require studies that demonstrates the signal and noise behavior impinging on the sensor. A statistical model has been introduced to study the signal and background noise and their effects on the SPAD. This analysis starts from the incoming photon flux, which depends on the optical system parameters (i.e., lens aperture, emitted pulse width, Field-of-View, optical efficiency) and optical power, and introduces some of the most important non-idealities (i.e., as hold-off time, afterpulsing, optical crosstalk and pile-up). Some acquisition techniques are also discussed as photon coincidence, time gating and multi- TDC. Every model is compared to the relative Monte Carlo simulation to highlight its quality. Finally, the reverse form of the model can be exploited to correct the pile-up distortion and estimate the incoming photon flux.

## References

[1] Wei X., Phung S.L., Bouzerdoum A. Pedestrian sensing using time-of-flight range camera. Proceedings of the IEEE Computer Vision and Pattern Recognition (CVPR 2011).

[2] Kollorz E., Penne J., Hornegger J., Barke A. (2008). Gesture recognition with a time-of-flight camera. Int. J. Intell. Syst. Technol. Appl..

[3] Cui Y., Schuon S., Chan D., Thrun S., Theobalt C. 3D shape scanning with a time-of-flight camera. Proceedings of the IEEE Computer Vision and Pattern Recognition (CVPR 2010).

[4] Donati S. (2004). Electro-Optical Instrumentation: Sensing and Measuring with Lasers.

[5] Villa F., Severini F., Madonini F., Zappa F. (2021). SPADs and SiPMs Arrays for Long-Range High-Speed Light Detection and Ranging (LiDAR). Sensors.

[6] BSI—British Standard (2007). BS EN 60825-1:2007—Safety of Laser Products. Equipment Classification and Requirements.

[7] Villa F., Lussana R., Bronzi D., Tisa S., Tosi A., Zappa F., Dalla Mora A., Contini D., Durini D., Weyers S. (2014). CMOS Imager With 1024 SPADs and TDCs for Single-Photon Timing and 3-D Time-of-Flight. IEEE J. Sel. Top. Quantum Electron..

[8] Buttafava M., Zeman J., Tosi A., Eliceiri K., Velten A. (2015). Non-line-of-sight imaging using a time-gated single photon avalanche diode. Opt. Express.

[9] Eshkoli A., Nemirovsky Y. (2021). Characterization and Architecture of Monolithic N⁺P-CMOS-SiPM Array for ToF Measurements. IEEE Trans. Instrum. Meas..

[10] Zappa F., Tosi A., Mora A.D., Tisa S. (2009). SPICE modeling of single photon avalanche diodes. Sens. Actuators A Phys..

[11] Villa F., Zou Y., Mora A.D., Tosi A., Zappa F. (2015). SPICE Electrical Models and Simulations of Silicon Photomultipliers. IEEE Trans. Nucl. Sci..

[12] Bronzi D., Zou Y., Villa F., Tisa S., Tosi A., Zappa F. (2016). Automotive Three-Dimensional Vision Through a Single-Photon Counting SPAD Camera. IEEE Trans. Intell. Transp. Syst..

[13] Sesta V., Severini F., Villa F., Lussana R., Zappa F., Nakamuro K., Matsui Y. (2021). Spot Tracking and TDC Sharing in SPAD Arrays for TOF LiDAR. Sensors.

[14] Kumagai O., Ohmachi J., Matsumura M., Yagi S., Tayu K., Amagawa K., Matsukawa T., Ozawa O., Hirono D., Shinozuka Y. A 189 × 600 Back-Illuminated Stacked SPAD Direct Time-of-Flight Depth Sensor for Automotive LiDAR Systems. Proceedings of the 2021 IEEE International Solid-State Circuits Conference (ISSCC).

[15] Zappa F., Tisa S., Tosi A., Cova S. (2007). Principles and features of single-photon avalanche diode arrays. Sens. Actuators A Phys..

[16] Tisa S., Zappa F., Tosi A., Cova S. (2007). Electronics for single photon avalanche diode arrays. Sens. Actuators A Phys..

[17] Ceccarelli F., Acconcia G., Gulinatti A., Ghioni M., Rech I., Osellame R. (2020). Recent Advances and Future Perspectives of Single-Photon Avalanche Diodes for Quantum Photonics Applications. Adv. Quantum Technol..

[18] Sarbazi E., Safari M., Haas H. (2018). Statistical Modeling of Single-Photon Avalanche Diode Receivers for Optical Wireless Communications. IEEE Trans. Commun..

[19] Padmanabhan P., Zhang C., Charbon E. (2019). Modeling and Analysis of a Direct Time-of-Flight Sensor Architecture for LiDAR Applications. Sensors.

[20] Sanzaro M., Gattari P., Villa F., Tosi A., Croce G., Zappa F. (2018). Single-Photon Avalanche Diodes in a 0.16 μm BCD Technology with Sharp Timing Response and Red-Enhanced Sensitivity. IEEE J. Sel. Top. Quantum Electron..

[21] Straka I., Grygar J., Hlousek J., Jezek M. (2020). Counting Statistics of Actively Quenched SPADs under Continuous Illumination. J. Light. Technol..

[22] Xu H., Pancheri L., Braga L.H., Dalla Betta G.F., Stoppa D. (2014). Crosstalk Characterization of Single-photon Avalanche Diode (SPAD) Arrays in CMOS 150nm Technology. Procedia Eng..

[23] Datta R., Heaster T.M., Sharick J.T., Gillette A.A., Skala M.C. (2020). Fluorescence lifetime imaging microscopy: Fundamentals and advances in instrumentation, analysis, and applications. J. Biomed. Opt..

[24] Scholkmann F., Kleiser S., Metz A.J., Zimmermann R., Pavia J.M., Wolf U., Wolf M. (2014). A review on continuous wave functional near-infrared spectroscopy and imaging instrumentation and methodology. NeuroImage.

[25] Tosi A., Zappa F., Cova S., Stellari F. (2003). Luminescence measurements for the investigation of VLSI circuit defects. Sens. Microsyst..

[26] Li Y., Ibanez-Guzman J. (2020). Lidar for Autonomous Driving: The principles, challenges, and trends for automotive lidar and perception systems. IEEE Signal Process. Mag..

[27] O’connor D., Philips D. (1984). Time-Correlated Single Photon Counting.

[28] GmbH P. (2014). Time-Correlated Single Photon Counting. http://www.picoquant.com/images/uploads/page/files/7253/technote_tcspc.pdf.

[29] Pediredla A.K., Sankaranarayanan A.C., Buttafava M., Tosi A., Veeraraghavan A. (2018). Signal Processing Based Pile-up Compensation for Gated Single-Photon Avalanche Diodes. arXiv.

[30] Beer M., Haase J.F., Ruskowski J., Kokozinski R. (2018). Background Light Rejection in SPAD-Based LiDAR Sensors by Adaptive Photon Coincidence Detection. Sensors.

[31] Arlt J., Tyndall D., Rae B.R., Li D.D.U., Richardson J.A., Henderson R.K. (2013). A study of pile-up in integrated time-correlated single photon counting systems. Rev. Sci. Instrum..

[32] Rapp J., Ma Y., Dawson R., Goyal V. (2021). High-flux single-photon lidar. Optica.

[33] Rapp J., Ma Y., Dawson R.M.A., Goyal V.K. (2019). Dead Time Compensation for High-Flux Ranging. IEEE Trans. Signal Process..

[34] Coates P.B. (1968). The correction for photonpile-up’ in the measurement of radiative lifetimes. J. Phys. E Sci. Instrum..

